# Implementation of locally processed neuron-specific enolase is associated with improved guideline-concordant neuroprognostication after out-of-hospital cardiac arrest: a retrospective before-and-after study

**DOI:** 10.1016/j.resplu.2026.101418

**Published:** 2026-07-17

**Authors:** Christopher D. Smith, Saxon Douglass, Nilesh A. Devanand, Daniel C. Adair, Samantha Nankivell, Megan Freeman, Philip Emerson, Peter Hibbert, Luke Collett, Krishnaswamy Sundararajan

**Affiliations:** a Intensive Care Medicine, Royal Adelaide Hospital, Adelaide, South Australia, Australia; b School of Medicine, Adelaide University, Adelaide, South Australia, Australia; c Department of Neurology, Alfred Hospital, Melbourne, Victoria, Australia; d SA Pathology, SA Health, Adelaide, South Australia, Australia; e Centre for Healthcare Resilience and Implementation Science, Australian Institute of Health Innovation, Macquarie University, Sydney, New South Wales, Australia; f IIMPACT in Health, Allied Health and Human Performance, Adelaide University, South Australia, Australia

**Keywords:** Neuron-specific enolase, Neuroprognostication, Out-of-hospital cardiac arrest, Quality improvement, Intensive care units

## Abstract

**Background:**

For comatose survivors of out-of-hospital cardiac arrest (OHCA), accurate neuroprognostication enables shared decision-making and avoids premature or unnecessarily delayed withdrawal of life-sustaining treatment. Guidelines recommend a multimodal approach requiring at least two independent poor-prognostic criteria, of which neuron-specific enolase (NSE) is currently the primary endorsed serum biomarker. NSE is not consistently used, and guideline adherence remains variable internationally.

**Objectives:**

To evaluate whether locally processed NSE improved guideline-concordant neuroprognostication and decision-making timelines after OHCA.

**Methods:**

A single-centre retrospective before-and-after study at an Australian quaternary intensive care unit (ICU) compared control (January 2022–July 2024) and intervention (August 2024–March 2026) periods. Sixty-nine comatose adults who underwent withdrawal of life-sustaining treatment on neurological grounds after OHCA were included. The primary outcome was guideline-concordant neuroprognostication (≥2 multimodal poor-prognostic criteria). Secondary outcomes were time to clinical determination of a poor neurological prognosis and prognostic disclosure to the family.

**Results:**

Guideline-concordant neuroprognostication increased from 35.9% (14/39) to 63.3% (19/30), an absolute increase of 27.4% (*p* = 0.030). Cumulative sum analysis showed sustained improvement throughout the intervention period. Time to clinical determination of a poor neurological prognosis (87.0 vs 88.4 h, *p* = 0.66) and prognostic disclosure to the family (91.6 vs 92.4 h, *p* = 0.69) were unchanged.

**Conclusions:**

Implementation of locally processed NSE was associated with a statistically significant increase in guideline-concordant multimodal neuroprognostication. Decision-making timelines were unchanged, consistent with prognostication being shaped by human and system-level factors beyond diagnostic availability. NSE availability was not associated with earlier withdrawal of life-sustaining treatment.

## Graphical abstract

**Figure:**
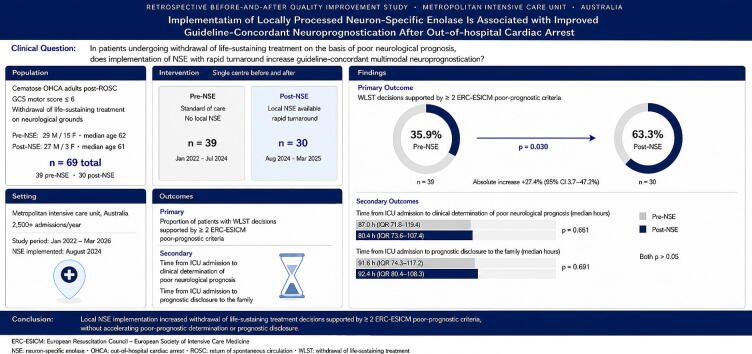

## Introduction

Out-of-hospital cardiac arrest (OHCA) is a leading cause of critical illness, with survival rates to hospital discharge ranging between 8% and 12% internationally.^1^ For patients who remain comatose after return of spontaneous circulation (ROSC), prognostic uncertainty creates significant challenges for clinicians and families alike.^2^ Timely and accurate neuroprognostication enables informed shared decision-making regarding goals of care and may avoid both premature withdrawal of life-sustaining treatment (WLST) and unnecessarily prolonged intensive care.^3^

The European Resuscitation Council (ERC) and European Society of Intensive Care Medicine (ESICM) guidelines, updated in 2025, recommend a multimodal approach to prognostication, with an unfavourable prognosis indicated by two independent poor-prognostic criteria after 72 h post-ROSC in comatose patients.^4^ These markers include absent pupillary and corneal reflexes at ≥72 h, highly malignant electroencephalography (EEG) patterns after 24 h, elevated neuron-specific enolase (NSE) levels (>60 µg/L) at 48 and/or 72 h, bilateral absent N20 waveforms on somatosensory evoked potentials (SSEP), status myoclonus within 72 h, and diffuse anoxic changes on neuroimaging (CT/MRI). These criteria have been prospectively validated with a near-zero false-positive rate across multiple studies in Europe and Asia.^5^, ^6^, ^7^ Conversely, WLST in patients with zero or one ERC-ESICM poor-prognostic criterion (indeterminate prognosis) risks a self-fulfilling prophecy: in a matched-cohort study from settings with minimal or no use of WLST in this context, up to 18% of comparable patients survived with good functional outcome when treatment was continued.^8^

Despite guideline recommendations, real-world adoption remains variable. Access to timely EEG and SSEP is limited in many intensive care units (ICUs).^9^ NSE, although validated as a biomarker of neuronal injury, remains inaccessible in a timely manner in many hospitals. It requires specialised processing, and turnaround times adequate for outpatient neuroendocrine tumour monitoring – the original use case for NSE – are not suitable for inpatient neuroprognostication. Internationally, neuroprognostication practices frequently deviate from established guideline recommendations, risking either false-positive diagnosis of poor prognosis or unnecessarily delayed WLST.^10^

Families require timely, consistent, and comprehensible communication about prognosis to enable informed participation in decisions, reduce uncertainty, and align care with the patient’s values.^11^, ^12^ Neuroprognostication must therefore be understood as both a diagnostic and a communicative process, integrating clinical data with discussions that support families in navigating decision-making during this critical period.

In August 2024, our quaternary metropolitan ICU implemented locally processed NSE with a turnaround time of 2 h, as part of a pragmatic quality improvement initiative supported by a structured education strategy.

## Objective

This study evaluated whether implementation of locally processed NSE improved adherence to guideline-based multimodal neuroprognostication and influenced decision-making timelines among patients who underwent WLST on neurological grounds after OHCA. The primary outcome was guideline-concordant neuroprognostication, defined as documentation of ≥2 ERC-ESICM poor-prognostic criteria supporting the WLST decision. Secondary outcomes were the time from ICU admission post-ROSC to clinical determination of a poor neurological prognosis and the time from ICU admission post-ROSC to prognostic disclosure to the family. Exploratory outcomes included institutional unit costs of prognostication modalities, which were summarised descriptively.

## Methods

### Study design

A single-centre retrospective before-and-after study was conducted at a quaternary metropolitan Australian ICU. Patients who underwent WLST on neurological grounds after OHCA were compared between a pre-implementation period, before locally processed NSE was available (January 2022–July 2024), and a post-implementation period, after local NSE implementation (August 2024–March 2026). Clinician practice was not mandated or protocolised; clinicians were informed of NSE availability, and its use was encouraged within the context of contemporary neuroprognostication guidance.

International guidance from the ERC-ESICM, American Heart Association, Neurocritical Care Society, Canadian Cardiovascular Society, and Australian and New Zealand Committee on Resuscitation (ANZCOR) consistently supports delayed, confounder-free, multimodal neuroprognostication after cardiac arrest, with caution against basing prognosis on any single finding in isolation.^2^, ^4^, ^13^, ^14^, ^15^ Guideline concordance in this study was assessed against the ERC-ESICM neuroprognostication algorithm because it provides an explicit, auditable threshold of ≥2 independent poor-prognostic criteria, has been externally validated, and was the framework already familiar to the department. Concordance was determined by study investigators using original investigation results and clinical documentation, with poor-prognostic criteria defined a priori.

### Ethical approval

The project was reviewed by the Central Adelaide Local Health Network Safety and Quality Unit. It was determined to meet criteria for quality improvement activity and was therefore exempt from full human research ethics committee review in accordance with institutional policy and the National Statement on Ethical Conduct in Human Research. Individual patient consent was waived as this study involved retrospective analysis of routinely collected, de-identified clinical data and posed negligible risk to participants.

### Setting

The study was conducted in a quaternary metropolitan academic referral centre admitting over 2500 patients annually, including approximately 130 OHCA survivors. Prior to August 2024, NSE was not routinely available for clinical neuroprognostication, with routine turnaround times exceeding the timeframe for clinical utility; the only pre-implementation results were obtained during the assay wash-in and validation period. EEG, SSEP, CT, and MRI were available with variable time to investigation and reporting.

### Participants

Adult patients (≥18 years) admitted following OHCA who remained comatose after ROSC, defined as not awake and not obeying commands (Glasgow Coma Scale [GCS] motor score <6), and in whom a decision was subsequently made to withdraw life-sustaining treatment on neurological grounds, were included. Patients who regained consciousness, were declared dead by neurological criteria, died before prognostication, or underwent WLST for other reasons (multi-organ failure, pre-existing comorbidity, or previously documented treatment limitations) were excluded.

### Intervention

From August 2024, NSE was made available for local laboratory processing with a turnaround time of 2 h. Clinical adoption was supported through a structured implementation strategy: (i) direct email communication to ICU clinicians regarding local availability, cost, and intended clinical application; (ii) targeted education at clinical governance meetings and trainee medical officer teaching sessions, outlining NSE’s role within the ERC-ESICM multimodal prognostication framework; and (iii) pragmatic framing by consultant and trainee change champions who promoted awareness without mandating NSE use. Data were collected under routine clinical conditions to reflect real-world implementation.

### Data collection and statistical analysis

A statewide electronic medical record system allowed review of prehospital, referring hospital and ICU documentation. Demographic, arrest-related, and neuroprognostication data were extracted from these records.

Data extraction was performed by study investigators using a predefined data collection template. Investigators were not blinded to study period or NSE implementation status, as allocation was determined by admission date in this retrospective before-and-after study. To reduce subjective classification, poor-prognostic criteria were defined a priori, NSE results were verified against the laboratory information system, and original radiology and neurophysiology reports were manually reviewed. An independent assessment of each criterion by a second independent clinician was undertaken in all 69 patients.

Routinely collected ICU variables, including illness severity, physiological and laboratory markers, ICU length of stay, ICU mortality, and hospital mortality, were obtained from locally held data submitted to the Australian and New Zealand Intensive Care Society Adult Patient Database (ANZICS APD). Neuroprognostication variables included Glasgow Coma Scale (GCS) motor score, pupillary and corneal reflexes, status myoclonus, EEG, SSEP, NSE at 48 and/or 72 h, and CT/MRI findings. EEG findings were reported using American Clinical Neurophysiology Society (ACNS) standardised critical care EEG terminology. Highly malignant EEG patterns were defined as suppressed background, with or without periodic discharges, or burst-suppression, consistent with international guidelines.^2^, ^4^, ^16^

Clinical examination criteria were adjudicated from the last documented neurological examination before clinical determination of a poor neurological prognosis, provided this occurred at or beyond the guideline-specified time point. Only poor-prognostic criteria documented or available before this determination were considered for guideline concordance.

Time to clinical determination of a poor neurological prognosis was defined as the time from ICU admission post-ROSC to the documented point at which the treating team concluded that prognosis was poor and formed the basis for WLST on neurological grounds. Time to prognostic disclosure to the family was defined as the time from ICU admission post-ROSC to the time of the first documented family discussion communicating certainty of poor neurological prognosis and/or plan for WLST. Cases in which either time point was undocumented or could not be established with certainty were recorded as ‘unknown’.

Institutional unit costs were collected and summarised descriptively; neuroimaging costs were determined from itemised charges from the on-site radiology service, EEG costs were estimated using 2025 South Australian government salary scales, and laboratory costs for NSE assays were obtained from on-site pathology services. No formal economic evaluation was undertaken.

A power calculation was performed using data from a hypothesis-generating pilot study following NSE implementation. To detect an estimated 30% absolute difference in guideline concordance, it was predicted that this study would require approximately 43 patients in each group (86 total) to provide 80% power at a two-sided significance level of 0.05.

Categorical variables were compared using Fisher’s exact test and are presented as counts and proportions. Continuous variables were assessed for normality using the Shapiro–Wilk test. Continuous timing outcomes were right-skewed and are presented as medians with interquartile ranges, with between-group comparisons performed using the Mann–Whitney *U* test. Analyses were performed using R (version 4.3.2).^17^ A cumulative sum (CUSUM) control chart was constructed to visualise temporal process change, with ±2 standard deviation control limits.^18^ Analyses used available-case data; one patient had missing time to clinical determination of poor neurological prognosis and two had missing time to prognostic disclosure to the family, all in the pre-implementation group.

SQUIRE 2.0 standards were used to plan, evaluate and report the study results.^19^ A completed SQUIRE 2.0 checklist is provided as a supplementary file.

## Results

### Cohort characteristics

**Fig. 1:**
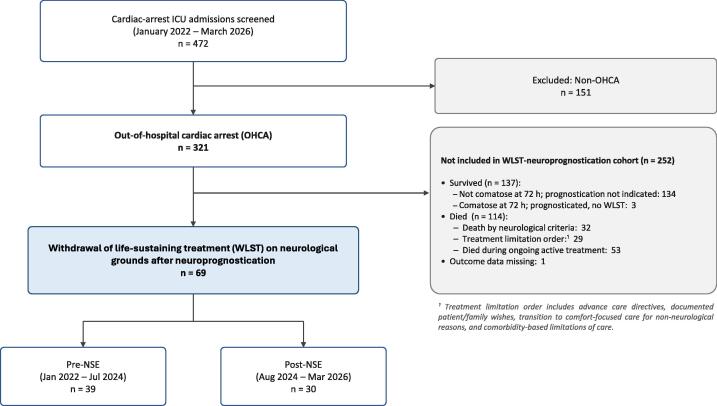
**Patient flow diagram for cohort selection**.

Between January 2022 and March 2026, 472 ICU admissions for cardiac arrest were screened using local ANZICS APD data, 321 of whom were OHCA. Of these, 69 progressed to withdrawal of life-sustaining treatment on the basis of neuroprognostication and met inclusion criteria: 39 in the pre-implementation period and 30 in the post-implementation period. Patients not included (*n* = 252) comprised those who survived to hospital discharge (137), were declared dead by neurological criteria (32), died from other causes, including before prognostication was feasible, in the context of multi-organ failure or advance care directives (82), or had no recorded outcome (1) (Fig. 1). One patient in the pre-NSE group met inclusion criteria while receiving extracorporeal membrane oxygenation.

Baseline characteristics were generally comparable, with differences in sex and presenting rhythm highlighted in Table 1. Presumed causes of arrest are summarised in Table 2. Median (IQR) APACHE III severity scores were 108 (96–125) pre-implementation and 110 (98–126) post-implementation, reflecting a uniformly critically ill cohort. Median (IQR) ICU length of stay was 4.6 (3.5–5.7) days versus 4.8 (4.0–5.8) days, respectively. In keeping with the inclusion criterion, in-hospital mortality approached 100% (68/69; Table 1). The single patient not recorded as dying in hospital was transferred to another facility for culturally appropriate end-of-life care following the decision to withdraw life-sustaining treatment.

**Table 1:** Cohort characteristics.

**Characteristic**	**Pre (*n* = 39)**	**Post (*n* = 30)**
***Demographics***		
Age, years	62.1 [50.4–69.1]	60.7 [46.2–68.5]
**Sex**		
Female	15/39 (38.5%)	3/30 (10.0%)
Male	24/39 (61.5%)	27/30 (90.0%)
First Nations peoples	3/39 (7.7%)	2/30 (6.7%)
***Arrest characteristics***		
Time to ROSC known/documented	34/39 (87.2%)	27/30 (90.0%)
Time to ROSC, min	25 [15–30]	25 [15–38]
**Initial rhythm**		
Shockable rhythm (VF/VT)	10/39 (25.6%)	14/30 (46.7%)
Non-shockable rhythm (PEA/asystole)	27/39 (69.2%)	15/30 (50.0%)
Other/unknown rhythm	2/39 (5.1%)	1/30 (3.3%)
***Physiology and illness severity***		
pH	7.25 [7.14–7.29]	7.25 [7.17–7.33]
Lactate, mmol/L	3.1 [2.2–6.4]	4.5 [2.6–5.9]
Lowest MAP, mmHg	61 [58–66]	65 [61–69]
Peak creatinine, µmol/L	100 [73–131] (*n* = 38)	118 [106–186]
Bilirubin, µmol/L	9 [6–12] (*n* = 37)	12 [8–19]
Lowest platelet count, ×10^9^/L	198 [175–247] (*n* = 37)	200 [156–232]
Lowest temperature, °C	35.1 [34.5–35.7]	35.1 [34.4–35.9]
APACHE III score	108 [96–125]	110 [98–126]
Invasive mechanical ventilation, *n* (%)	39/39 (100%)	30/30 (100%)
***ICU course and outcome***		
ICU length of stay, days	4.6 [3.5–5.7]	4.8 [4.0–5.8]
Died in ICU	36/39 (92.3%)	27/30 (90.0%)
Died in hospital	38/39 (97.4%)*	30/30 (100.0%)

Values are median [IQR] or *n*/*N* (%) unless otherwise stated; (*n* = …) indicates missing data. APACHE III, Acute Physiology and Chronic Health Evaluation III; ICU, intensive care unit; IQR, interquartile range; MAP, mean arterial pressure; NSE, neuron-specific enolase; PEA, pulseless electrical activity; ROSC, return of spontaneous circulation; VF, ventricular fibrillation; VT, ventricular tachycardia.

* 1 patient transferred out of hospital for culturally appropriate end-of-life care.

**Table 2:** Cause of cardiac arrest.

**Presumed cause of arrest**	**Pre (*n* = 39)**	**Post (*n* = 30)**
Cardiac/presumed cardiac	15/39 (38.5%)	16/30 (53.3%)
Respiratory, hypoxia or asphyxia	11/39 (28.2%)	6/30 (20.0%)
Pulmonary embolus/obstructive	4/39 (10.3%)	1/30 (3.3%)
Other medical non-cardiac*	3/39 (7.7%)	2/30 (6.7%)
Toxicological/overdose	1/39 (2.6%)	1/30 (3.3%)
Trauma	1/39 (2.6%)	0/30 (0.0%)
Unknown/not established	4/39 (10.3%)	4/30 (13.3%)

* Includes neurological, metabolic and electrolyte disturbance and shock states.

### NSE implementation and uptake

Following NSE implementation, at least one NSE sample was obtained in 28 of 30 post-NSE patients (93.3%) between 48 and 72 h, confirming near-universal and sustained adoption of local biomarker processing (Table 3). Of the 28 patients with at least one sample, NSE exceeded the ERC-ESICM threshold of 60 µg/L at one or both time points in 26 of 28 (92.9%). Seven patients with elevated NSE did not meet the threshold for guideline-concordant prognostication because no second independent poor-prognostic criterion was documented. In these seven patients, additional modalities were either not performed/not documented or, where performed, did not meet poor-prognostic thresholds (Table 3). By contrast, NSE was measured in only 3 of 39 pre-implementation patients (7.7%), during the assay wash-in and validation period preceding local go-live; in 2, above-threshold results were reported and available to the treating team before prognostic determination and contributed to guideline-concordance classification in that cohort.

**Table 3:** Neuroprognostication modalities and poor-prognostic findings.

**Domain**	**Modality**	**Pre (*n* = 39)**	**Post (*n* = 30)**	**Positive finding definition**
Clinical	*Pupillary reflex assessed/absent*	*39/3*	*30/3*	*Bilateral absent pupillary reflex*
	*Corneal reflex assessed/absent*	*16/4*	*11/3*	*Bilateral absent corneal reflex*
	**Both pupillary and corneal reflexes assessed/both absent**	**16/3**	**11/2**	**Bilateral absence of both pupillary and corneal reflexes**
	**Status myoclonus present**	**9**	**6**	**Status myoclonus documented**
Electrophysiology	**EEG performed/highly malignant EEG**	**18/13**	**11/6**	**Highly malignant EEG pattern –** suppressed background, with or without periodic discharges, or burst-suppression
	**SSEP performed/bilaterally absent N20 responses**	**1/0**	**0/0**	**Bilaterally absent N20 response**
Biochemical	**NSE measured/>60 µg/L at 48 h and/or 72 h**	**3/2**	**28/26**	**NSE >60 µg/L at 48 and/or 72 h**
Imaging	**CT or MRI performed/diffuse anoxic injury**	**36/16**	**24/13**	**Diffuse anoxic injury on either CT or MRI**
	*CT performed/diffuse anoxic injury*	*36/13*	*21/9*	*Diffuse anoxic injury on CT*
	*MRI performed/diffuse anoxic injury*	*5/5*	*6/4*	*Diffuse anoxic injury on MRI*

Values are n assessed or performed/*n* positive. Bold rows indicate the ERC-ESICM poor-prognostic criteria used to determine guideline-concordant neuroprognostication; non-bold subsidiary rows provide component or modality-specific detail. Status myoclonus values represent *n* documented as present.

ACNS, American Clinical Neurophysiology Society; CT, computed tomography; EEG, electroencephalography; ERC-ESICM, European Resuscitation Council and European Society of Intensive Care Medicine; MRI, magnetic resonance imaging; NSE, neuron-specific enolase; SSEP, somatosensory evoked potentials.

### Primary outcome: guideline-concordant neuroprognostication

The proportion of patients receiving guideline-concordant neuroprognostication (≥2 ERC-ESICM poor-prognostic criteria) increased from 35.9% (14/39) in the pre-implementation cohort to 63.3% (19/30) in the post-implementation cohort (absolute increase +27.4%; Fisher’s exact test *p* = 0.030; Table 4).

**Table 4:** Primary and secondary outcomes.

**Outcome**	**Pre-NSE (*n* = *39*)**	**Post-NSE (*n* = *30*)**	***p*-value**
***Primary outcome***			
Guideline-concordant neuroprognostication^a^, *n* (%)	14/39 (35.9%)	19/30 (63.3%)	0.030^b^
*Absolute difference*	–	+27.4% (95% CI 3.7–47.2%)	–

***Secondary outcomes***			
Time to determination of poor neurological prognosis, median (IQR), h	87.0 (71.8–111.9)	88.4 (73.6–107.4)	0.661^c^
Time to prognostic disclosure to the family, median (IQR), h	91.6 (74.3–117.3)	92.4 (80.4–108.3)	0.691^c^

CI, confidence interval; IQR, interquartile range; NSE, neuron-specific enolase; WLST, withdrawal of life-sustaining treatment.

a Guideline-concordant neuroprognostication was defined as documentation of ≥2 ERC-ESICM poor-prognostic criteria supporting the WLST decision.

b Fisher’s exact test (two-tailed).

c Mann–Whitney *U* test (two-tailed). Continuous timing outcomes were non-normally distributed; medians and interquartile ranges are reported.

**Fig. 2:**
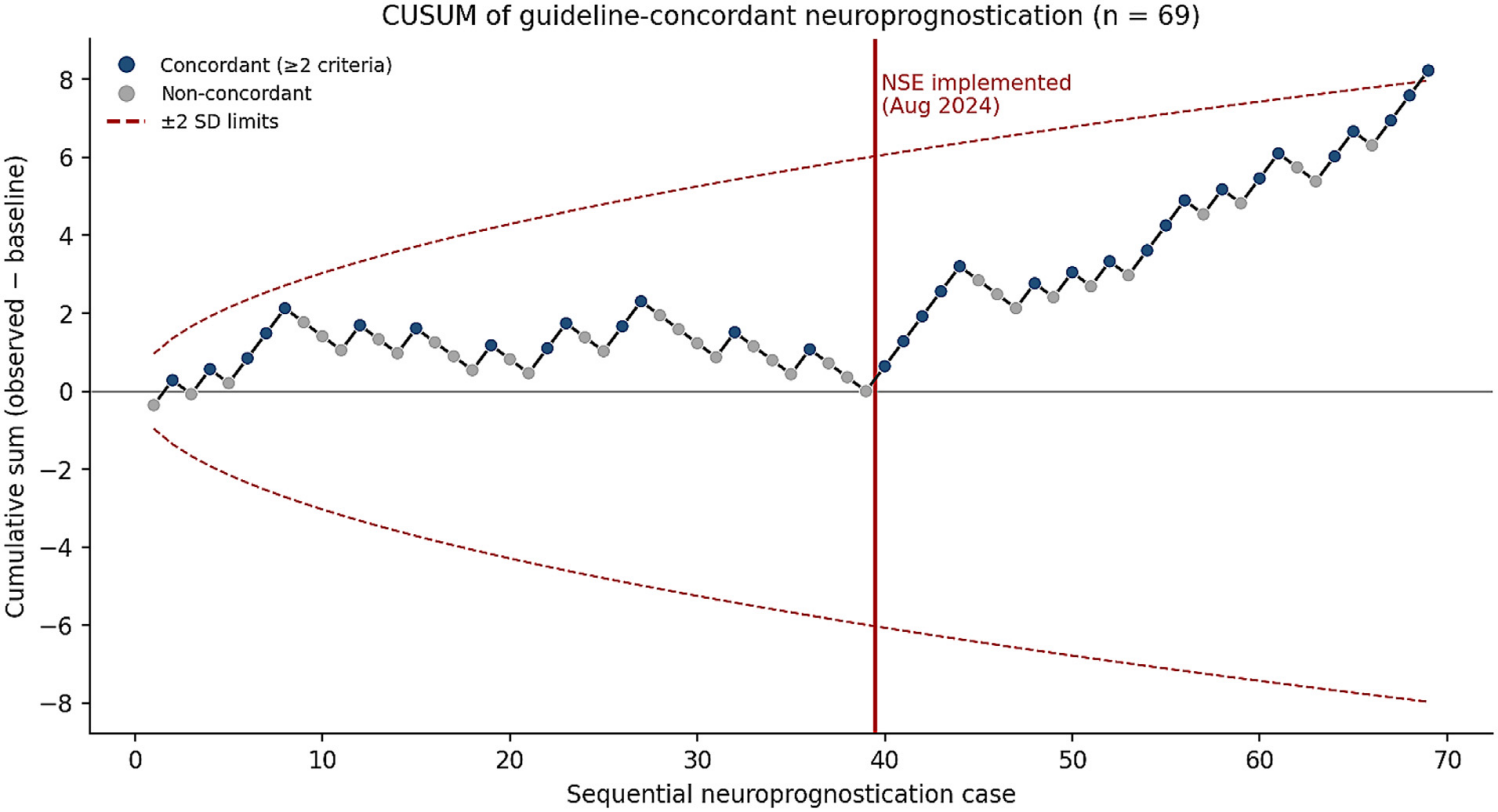
**Cumulative sum (CUSUM) control chart of sequential guideline-concordant neuroprognostication across the study period**. Each point represents a sequential patient case, coloured by whether guideline-concordant neuroprognostication (≥2 ERC-ESICM poor-prognostic criteria) was achieved (blue) or not (grey). The red vertical line denotes NSE implementation (August 2024). Cases are ordered by ICU admission date. The horizontal line at zero represents the pre-implementation baseline concordance rate (35.9%); the widening red dashed lines are the ±2 standard-deviation control limits. The cumulative sum remained within these limits throughout, reaching the upper limit at the final case. (For interpretation of the references to colour in this figure legend, the reader is referred to the web version of this article.)

Cumulative sum (CUSUM) analysis monitored sequential patient-level changes in guideline adherence, ordered by ICU admission date and using the pre-implementation adherence rate of 35.9% as the reference baseline (Fig. 2). During the pre-implementation phase, the CUSUM trajectory was flat to marginally negative, consistent with process stability. Following NSE implementation, the CUSUM rose progressively across all post-implementation cases, reaching the upper ±2 SD control limit by the end of the observation period. This pattern indicates a consistent shift in process performance above the pre-implementation baseline.

### Secondary outcomes

**Fig. 3:**
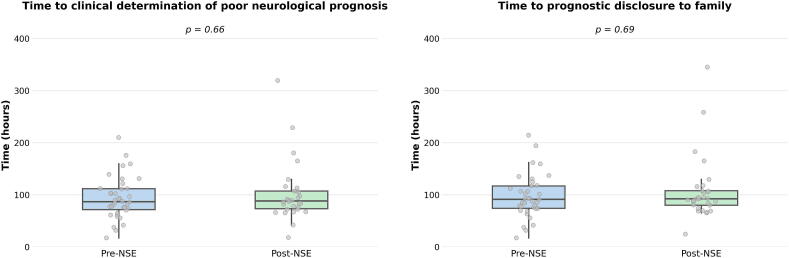
**Box-plot demonstrating time to clinical determination of a poor neurological prognosis and prognostic disclosure to the family pre- and post-implementation of rapid turnaround neuron-specific enolase**. Boxes represent IQR, centre lines medians, whiskers 1.5 × IQR, and points individual observations.

There was no statistically significant difference in the time from ICU admission post-ROSC to documented clinical determination of a poor neurological prognosis between groups: median (IQR) pre-implementation 87.0 (71.8–111.9) h versus post-implementation 88.4 (73.6–107.4) h (Mann–Whitney U, *p* = 0.66). Time to prognostic disclosure to the family was also not significantly different: median (IQR) pre-implementation 91.6 (74.3–117.3) h versus post-implementation 92.4 (80.4–108.3) h (*p* = 0.69; Fig. 3, Table 4). Both distributions were right-skewed, with outliers reflecting complex cases with prolonged diagnostic uncertainty or family discussion in both cohorts.

### Cost context

For contextual reference, the institutional unit cost of NSE was AUD $21.60 per assay, compared with AUD $188.15 for EEG, AUD $378.50 for CT brain, and AUD $569.63 for MRI brain. These figures are presented as institutional unit costs only and do not constitute a formal economic analysis.

## Discussion

### Principal findings

In this four-year retrospective before-and-after study of consecutive OHCA admissions in which life-sustaining treatment was withdrawn on the basis of poor neurological prognosis, the introduction of locally processed NSE with rapid (2 h) turnaround was associated with a statistically significant increase in guideline-concordant neuroprognostication. The proportion of comatose post-arrest patients receiving ≥2 ERC-ESICM poor-prognostic criteria increased from 35.9% to 63.3% following implementation, an absolute improvement of 27.4%. CUSUM analysis demonstrated a sustained upward trajectory above the pre-implementation baseline. Median times to clinical determination of a poor neurological prognosis and prognostic disclosure to the family remained unchanged at 87-92 h after ICU admission.

### Why diagnostic access matters

These findings suggest that diagnostic accessibility is a modifiable barrier to neuroprognostication guideline adherence. NSE provides objective, quantitative evidence of neuronal injury that complements clinical examination, neurophysiological studies, and neuroimaging, and is rapidly available. Before NSE implementation, the majority of patients in our cohort received prognostication based on fewer than two positive criteria, most commonly clinical examination alone or in combination with non-confirmatory neuroimaging or electrophysiological studies. Post-implementation, the majority of patients met ERC-ESICM criteria for guideline-concordant prognostication at time of WLST. As guideline-discordant withdrawal is most consequential in patients with an indeterminate prognosis, improved concordance may reduce the risk of premature withdrawal in those with the potential for a good functional outcome.

These findings add Australasian real-world evidence to a literature consistently showing that compliance with international guidance remains variable years after publication. Whereas the prognostic validity of NSE is well established and its variable uptake documented, the effect of improving timely local access to the biomarker on guideline-concordant practice has received little attention. Our data add before-and-after evidence that addressing access to this biomarker with rapid turnaround translates into meaningful improvement in guideline adherence.

### Why decision-making timelines did not change

The stability of the time to clinical determination of a poor neurological prognosis and prognostic disclosure to the family, despite near-universal NSE use and a high rate of positive results, is informative. It reflects that prognostication is a communicative and interpersonal process, not only a diagnostic one. Clinical caution and emotional complexity frequently delay discussions about poor prognosis and withdrawal of organ support, even when prognostic evidence is present. Clinicians must achieve consensus, families must process devastating information, and discussions must be appropriately timed to align with the patient’s values and the family’s readiness. These factors are governed by human and organisational dynamics rather than clinician diagnostic certainty alone.

Our data suggest that median decision-making timelines of approximately 87‒92 h after ICU admission reflect appropriate clinical practice at our institution, consistent with international recommendations that formal prognostication should not begin until at least 72 h post-ROSC. While structured prognostication protocols improve consistency, they do not necessarily expedite decisions about the withdrawal of life-sustaining treatment.^20^ Critically and reassuringly, NSE availability and use at 48 h was not associated with premature prognostication.

Although the median time from ICU admission to clinical determination exceeded 72 h, 16 of the 68 patients with a recorded time (24%; 10/38 pre- and 6/30 post-implementation) had their determination documented within 72 h of admission, in some cases considerably earlier. Because the available data allowed timing to be measured only from ICU admission rather than from ROSC, this should not be read as a direct assessment of the guideline-recommended 72-h post-ROSC prognostication window; in retrieved patients, ICU admission may have occurred a significant time after ROSC. Nevertheless, timing represents a dimension of guideline concordance distinct from the multimodal requirement for ≥2 criteria assessed in the primary outcome, and one the intervention did not directly address. Early prognostic determination and withdrawal of life-sustaining treatment carry a heightened risk of self-fulfilling prophecy, particularly in patients with an indeterminate prognosis, and 9 of these 16 determinations were in patients who did not meet ≥2 criteria. The availability of NSE at 48 h was not associated with an increase in determinations within 72 h of ICU admission (20% post- versus 26% pre-implementation), although the persistence of earlier decisions in both cohorts identifies a priority for future work.

### Strengths and limitations

The strengths of this study include its pragmatic real-world design, extended observation period spanning four years, and 100% consecutive case inclusion within the WLST cohort. NSE uptake of 93% in the post-implementation period confirms the intervention was delivered as intended. Combining CUSUM with inferential statistics provides temporal context that cross-sectional comparisons cannot, supporting a sustained rather than transient change.

Several limitations must be acknowledged. The study is single-centre, limiting generalisability despite representing a quaternary referral centre serving a large Australian region. The WLST post-neuroprognostication cohort remains relatively small (*n* = 69), reflecting the uncommon intersection of OHCA, sustained coma, and prognostication at a single institution. The enrolled cohort also fell short of the pre-specified target of 86 patients. NSE use was promoted but not mandated, and educational effects may wane without embedded protocol integration. Given the retrospective design, unmeasured confounders, including changes in case mix, staffing, or concurrent practice, cannot be excluded. By design, the cohort was restricted to patients who underwent WLST on neurological grounds. This allowed evaluation of whether WLST decisions were supported by guideline-concordant multimodal neuroprognostication, but limits inference about prognostic accuracy, false-positive rates, survival, or neurological outcomes among all comatose OHCA survivors. Finally, the dataset does not capture neurological outcomes beyond ICU admission and cannot determine whether improved guideline adherence changes patient outcomes.

### Implications for practice and research

These findings support adoption of locally processed NSE as a feasible, low-cost component of multimodal neuroprognostication that can meaningfully improve guideline adherence. For hospitals where NSE is unavailable or reliant on external processing, local implementation with a target turnaround of 2 h is an achievable quality improvement initiative. As an exploratory observation, NSE was the least costly of the prognostication modalities used at our institution (AUD $21.60 per assay); comparative EEG and imaging costs are reported in the Cost Context section. While no formal cost-effectiveness analysis was conducted, this cost context may be relevant to health services considering local biomarker implementation. Full economic evaluation, including laboratory infrastructure, clinician time, and downstream decision-making costs, would be required before cost-effectiveness conclusions could be drawn.

Diagnostic access alone is insufficient to optimise neuroprognostication practice. Health services should consider embedding guideline-aligned requirements for ≥2 independent poor-prognostic criteria into local practice, establishing key performance indicators for NSE turnaround time and sampling at 48 and 72 h post-ROSC, and implementing regular audit-and-feedback cycles. Grounding withdrawal decisions in guideline-concordant multimodal evidence may reduce the risk of premature withdrawal when prognosis is uncertain.

Future research should include multicentre, system-level studies assessing whether governance-integrated approaches improve guideline concordance and patient- and family-centred outcomes, alongside qualitative work exploring clinician and family perspectives on the barriers to timely prognostic conversations.^21^

## Conclusions

In this four-year retrospective cohort study, implementation of locally processed NSE at a quaternary Australian ICU was associated with a statistically significant increase in guideline-concordant multimodal neuroprognostication following OHCA, increasing from 35.9% to 63.3% (*p* = 0.030). Decision-making timelines remained stable, consistent with the understanding that prognostication is shaped by human and system-level factors beyond diagnostic availability.

## CRediT authorship contribution statement

**Christopher D. Smith:** Writing – review & editing, Writing – original draft, Visualization, Supervision, Project administration, Methodology, Investigation, Data curation, Conceptualization. **Saxon Douglass:** Writing – review & editing, Methodology, Investigation, Formal analysis, Conceptualization. **Nilesh A. Devanand:** Writing – review & editing, Writing – original draft, Visualization. **Daniel C. Adair:** Investigation, Data curation. **Samantha Nankivell:** Resources, Conceptualization. **Megan Freeman:** Resources, Investigation. **Philip Emerson:** Writing – review & editing, Visualization, Validation, Methodology. **Peter Hibbert:** Writing – review & editing, Supervision, Methodology. **Luke Collett:** Supervision, Investigation, Formal analysis, Conceptualization. **Krishnaswamy Sundararajan:** Writing – review & editing, Supervision.

## Ethics approval

This study was reviewed by the Central Adelaide Local Health Network Safety and Quality Unit and determined to meet criteria for quality improvement activity, exempt from full human research ethics committee review in accordance with institutional policy and the National Statement on Ethical Conduct in Human Research. All interventions were compliant with relevant institutional guidelines and the Declaration of Helsinki.

## Funding

This study received no funding support.

## Data availability

Data are available upon reasonable request, subject to approval from the relevant research ethics committee.

## Declaration of Generative AI use

Artificial intelligence-based tools were used during the preparation of this manuscript to assist with language refinement, structural organisation, and editorial clarity. No artificial intelligence system was used to generate original scientific content, interpret data, or make clinical judgements. All content was reviewed, verified, and approved by the authors, who take full responsibility for the accuracy and integrity of the manuscript.

## Declaration of competing interest

The authors declare that they have no known competing financial interests or personal relationships that could have appeared to influence the work reported in this paper.

## Appendix A Supplementary material

Supplementary Data 1 SQUIRE_{20}_Checklist.
