# Supplementary material for: Implementation of locally processed neuron-specific enolase is associated with improved guideline-concordant neuroprognostication after out-of-hospital cardiac arrest: a retrospective before-and-after study
Source: Resusc Plus. 2026 Jul 17;30:101418. doi: 10.1016/j.resplu.2026.101418 (PMC13450595; doi:10.1016/j.resplu.2026.101418)
Supplement: Supplementary file 1 [file mmc1.docx]

**SQUIRE 2.0 Checklist**

Implementation of Locally Processed Neuron-Specific Enolase Is Associated with Improved Guideline-Concordant Neuroprognostication After Out-of-Hospital Cardiac Arrest: A Retrospective Before-and-After Study

| **Item** | **Name** | **Description** | **Location in manuscript** |
| --- | --- | --- | --- |
| **Title and Abstract** | | | |
| 1 | **Title** | Indicates initiative to improve healthcare | Title page |
| 2a | **Abstract – searchability** | Adequate information for searching and indexing | Abstract |
| 2b | **Abstract – structure** | Background, local problem, methods, interventions, results, conclusions | Abstract |
| **Introduction — Why did you start?** | | | |
| 3 | **Problem description** | Nature and significance of the local problem (variable NSE access; guideline non-adherence) | Introduction, paragraphs 1–3 |
| 4 | **Available knowledge** | Summary of current evidence on NSE and neuroprognostication guideline adherence | Introduction, paragraphs 2–4 |
| 5 | **Rationale** | Diagnostic accessibility as a modifiable barrier; rationale for local NSE processing | Introduction, paragraph 4; Discussion |
| 6 | **Specific aims** | Primary and secondary outcomes stated | Objective section |
| **Methods — What did you do?** | | | |
| 7 | **Context** | Quaternary academic ICU; ~130 OHCA survivors/year; prior absence of local NSE | Methods: Setting |
| 8a | **Intervention – description** | Local NSE processing, <12h TAT; three-part implementation strategy described | Methods: Intervention |
| 8b | **Intervention – team** | Consultant and registrar change champions; education at governance meetings | Methods: Intervention |
| 9a | **Study of intervention – approach** | Retrospective before-and-after cohort design | Methods: Study Design |
| 9b | **Study of intervention – attribution** | Temporal attribution via CUSUM; pre-post comparison; limitation of confounding acknowledged | Methods: Study Design; Discussion: Limitations |
| 10a | **Measures – outcomes** | Guideline-concordant neuroprognostication (≥2 ERC-ESICM criteria); time to clinical determination of a poor neurological prognosis; time to prognostic disclosure to the family | Methods: Data Collection; Objective section |
| 10b | **Measures – context** | Institutional costs reported as exploratory contextual observation | Methods: Data Collection; Discussion |
| 10c | **Measures – data accuracy** | Data extracted from the statewide electronic medical record; poor-prognostic criteria defined a priori, NSE verified against the laboratory information system, original radiology and neurophysiology reports reviewed, and each criterion independently assessed by a second clinician | Methods: Data Collection |
| 11a | **Analysis – methods** | Fisher's exact test; Mann–Whitney U; Shapiro–Wilk normality testing; R v4.3.2 | Methods: Data Collection and Statistical Analysis |
| 11b | **Analysis – variation over time** | CUSUM control chart with ±2 SD control limits for temporal process monitoring | Methods: Data Collection and Statistical Analysis; Results: Primary Outcome |
| 12 | **Ethical considerations** | Reviewed by CALHN Safety and Quality Unit; QI exemption; patient consent waived; retrospective de-identified data; Declaration of Helsinki compliance | Methods: Ethical Approval |
| **Results — What did you find?** | | | |
| 13a | **Results – intervention evolution** | NSE implemented August 2024; 93% uptake (28/30 sampled at 48 and/or 72 h) from outset with no protocol mandation | Results: NSE Implementation and Uptake |
| 13b | **Results – process and outcomes** | Primary and secondary outcomes reported with statistics; CUSUM trajectory described | Results: Primary Outcome; Secondary Outcomes |
| 13c | **Results – contextual elements** | Educational strategy and change champions described; pragmatic non-mandated adoption noted | Methods: Intervention; Discussion |
| 13d | **Results – associations** | NSE implementation associated with increased guideline concordance; no change in decision timelines | Results: Primary Outcome; Secondary Outcomes |
| 13e | **Results – unintended consequences** | No premature prognostication observed; cost context reported; no adverse unintended consequences identified | Discussion: Why Decision-Making Timelines Did Not Change |
| 13f | **Results – missing data** | One patient missing time to clinical determination of poor neurological prognosis; two missing time to prognostic disclosure to the family, all pre-implementation; available-case analysis used | Methods: Data Collection and Statistical Analysis |
| **Discussion — What does it mean?** | | | |
| 14a | **Summary – findings** | 27.4% absolute increase in guideline concordance; unchanged decision timelines | Discussion: Principal Findings |
| 14b | **Summary – strengths** | Pragmatic design; 4-year observation; 93% intervention uptake; CUSUM methodology | Discussion: Strengths and Limitations |
| 15a | **Interpretation – association** | Diagnostic access identified as modifiable barrier; NSE as low-cost complementary tool | Discussion: Why Diagnostic Access Matters |
| 15b | **Interpretation – comparison** | Consistent with international literature on guideline variability; first before-and-after NSE implementation study | Discussion: Why Diagnostic Access Matters |
| 15c | **Interpretation – impact** | Meaningful improvement in QI process; unchanged timelines reflect appropriate clinical practice | Discussion: Principal Findings; Why Decision-Making Timelines Did Not Change |
| 15d | **Interpretation – differences from anticipated** | Null result for decision timelines explained by communicative/relational prognostication factors | Discussion: Why Decision-Making Timelines Did Not Change |
| 15e | **Interpretation – costs** | Unit costs reported (NSE AUD $21.60 vs EEG $188.15, CT $378.50, MRI $569.63); no formal cost-effectiveness analysis conducted | Discussion: Implications for Practice and Research |
| 16a | **Limitations – generalisability** | Single-centre; small cohort (n=69); quaternary referral setting | Discussion: Strengths and Limitations |
| 16b | **Limitations – internal validity** | Retrospective design; unmeasured confounders; guideline update as potential co-intervention; educational effects may wane | Discussion: Strengths and Limitations |
| 16c | **Limitations – minimisation** | Consecutive case inclusion; CUSUM for temporal monitoring; available-case analysis | Methods; Discussion: Strengths and Limitations |
| 17a | **Conclusions – usefulness** | NSE feasible, low-cost, and associated with meaningful improvement in guideline adherence | Conclusions |
| 17b | **Conclusions – sustainability** | Uptake sustained without mandation; protocol embedding recommended for durability | Discussion: Implications for Practice and Research |
| 17c | **Conclusions – spread** | Applicable to quaternary and regional ICUs lacking local NSE; implementation strategy described for replication | Discussion: Implications for Practice and Research |
| 17d–e | **Conclusions – implications and next steps** | Multicentre studies; governance-integrated audit-and-feedback; qualitative research on barriers | Discussion: Implications for Practice and Research |
| **Other information** | | | |
| 18 | **Funding** | No funding received | Declarations: Funding |
